# Lack of association of *CFD* polymorphisms with advanced age-related macular degeneration

**Published:** 2010-11-03

**Authors:** Jiexi Zeng, Yuhong Chen, Zongzhong Tong, Xinrong Zhou, Chao Zhao, Kevin Wang, Guy Hughes, Daniel Kasuga, Matthew Bedell, Clara Lee, Henry Ferreyra, Igor Kozak, Weldon Haw, Jean Guan, Robert Shaw, William Stevenson, Paul D. Weishaar, Mark H. Nelson, Luosheng Tang, Kang Zhang

**Affiliations:** 1 Department of Ophthalmology, Second Xiangya Hospital, Central South University, Changsha, China; 2 Department of Ophthalmology & Vision Science, Eye and ENT hospital, Shanghai Medical School, Fudan University, Shanghai, China; 3 Institute for Genomic Medicine and Shiley Eye Center, University of California San Diego, San Diego, CA; 4 Department of Ophthalmology and Visual Sciences, Moran Eye Center, University of Utah School of Medicine, Salt Lake City, UT; 5 Vitreo-Retinal Consultants & Surgeons, 530 North Lorraine, Wichita, KS; 6 North Carolina Macular Consultants, Winston-Salem, NC

## Abstract

**Purpose:**

Age-related macular degeneration (AMD) is the most common cause of irreversible central vision loss worldwide. Research has linked AMD susceptibility with dysregulation of the complement cascade. Typically, complement factor H (*CFH*), complement factor B (*CFB*), complement component 2 (*C2*), and complement component 3 (*C3*) are associated with AMD. In this paper, we investigated the association between complement factor D (*CFD*), another factor of the complement system, and advanced AMD in a Caucasian population.

**Methods:**

Six single nucleotide polymorphisms (SNPs), rs1683564, rs35186399, rs1683563, rs3826945, rs34337649, and rs1651896, across the region covering *CFD,* were chosen for this study. One hundred and seventy-eight patients with advanced AMD and 161 age-matched normal controls were genotyped. Potential positive signals were further tested in another independent 445 advanced AMD patients and 190 controls. χ^2^ tests were performed to compare the allele frequencies between case and control groups.

**Results:**

None of the six SNPs of *CFD* was found to be significantly associated with advanced AMD in our study.

**Conclusions:**

Our findings suggest that *CFD* may not play a major role in the genetic susceptibility to AMD because no association was found between the six SNPs analyzed in the *CFD* region and advanced AMD.

## Introduction

Age-related macular degeneration (AMD) is the most common cause of irreversible central vision loss worldwide [1]. It can be divided into early and advanced (late) forms. Early AMD is characterized by the presence of drusen or pigmentary abnormalities in the retinal pigment epithelium (RPE), while advanced AMD includes two clinical types—geographic atrophy of the RPE (dry AMD) and choroidal neovascularization (wet AMD). Age-related macular degeneration is a complex disease caused by the combination of genetic predisposition and environmental factors [2-4]. The inheritance of AMD is polygenic and multifactorial: race, age, smoking, body mass, and body mass index have all been associated with AMD [2,5-9]. In the past five years, significant progress has been made in our understanding of AMD genetics through studies that identified specific single nucleotide polymorphisms (SNPs) significantly associated with AMD. The discovery of the strongest associations of AMD with the variants of complement factor H (*CFH*; OMIM 134370) [10-13] and *ARMS2/HTRA1* (age-related maculopathy susceptibility 2, OMIM 611313; the high temperature requirement factor A1, OMIM: 602194) [14-17] has led to new hypotheses regarding the pathogenesis of this disease. *CFH* is primarily associated with the formation of drusen that often characterizes both types of advanced AMD in Caucasian populations, whereas *ARMS2/HTRA1* is mainly associated with wet AMD [18]. Other than these two major loci, three other members of the complement system, complement component 2 (*C2*; OMIM 217000), complement component 3 (*C3*; OMIM 120700), and complement factor B (*CFB*; OMIM 138470), were also found to be associated with AMD [10,19-21]. Most of these genes are complement pathway-associated genes, with the products participating in the alternative complement pathway and playing important roles in the complement system. Many studies have provided evidence that the complement system is involved in the pathogenesis of AMD and may have a pivotal effect on the formation and development of the disease [13,22-24]. However, the specific role of the complement system in the etiology of AMD is difficult to elucidate, and questions remain as to whether other components from this system are associated with AMD.

Complement factor D (CFD) is a protein of the trypsin family encoded by the *CFD* gene (OMIM 134350) and is involved in the alternative complement pathway of the complement system [25,26]. CFD is unique among serine proteases in that it requires neither enzymatic cleavage for expression of proteolytic activity nor inactivation by a serpin for its control [27]. It is best known for its role in humoral suppression of infectious agents and has a high level of expression in fat, suggesting a role for adipose tissue in immune system biology (CFD). In this study, we investigated the association of *CFD* with advanced AMD in a Caucasian population.

## Methods

### Subjects and clinical diagnosis

This study was approved by the Institutional Review Board of the University of California, San Diego. Informed consent was signed by all subjects before participation in the study. One hundred and seventy-eight nonfamilial advanced AMD patients and 161 age-matched normal controls (60 years or older with no drusen or RPE changes) as well as an independent replication cohort with 445 nonfamilial advanced AMD patients and 190 age-matched normal controls were recruited using the standard ophthalmic examination protocol. Grading was performed using a standard grid classification suggested by the International Age-related Maculopathy (ARM) Epidemiological Study Group for the age-related maculopathy and age-related macular degeneration group [28]. All the participants were Caucasian.

### Genotyping

**Figure 1:**
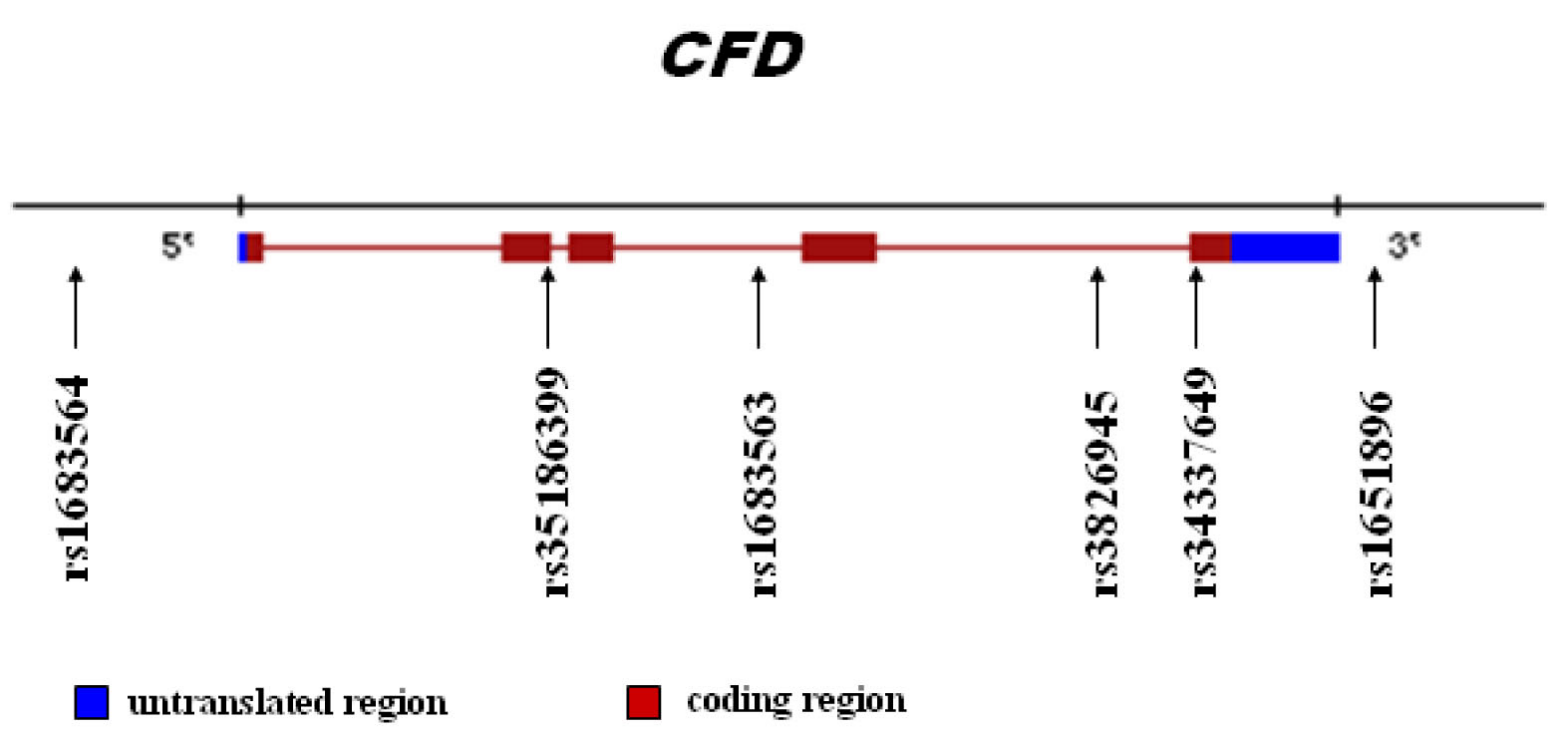
Six single nucleotide polymorphisms (SNPs) on complement factor D (*CFD).* Shown is the corresponding location of each SNP on *CFD* (chromosome:19; location:19p13.3) that was chosen in this study.

Six SNPs, rs1683564, rs35186399, rs1683563, rs3826945, rs34337649, and rs1651896 at the *CFD* locus were chosen, which were either tag SNPs or those that might affect the function of *CFD* (Figure 1). A Caucasian cohort of 178 advanced AMD patients was genotyped and allele frequencies were compared with 161 age- and ethnicity-matched normal controls by laboratory personnel blinded to the case/control status. Potential positive findings were tested for replication in an independent cohort of 445 advanced AMD patients and 190 normal controls.

Genotypes of six SNPs were achieved by primer extension of multiplex PCR products followed with a SNaPshot using an ABI 3100 genetic analyzer (Applied Biosystems, Foster City, CA).

### Statistical analysis

Deviation from Hardy–Weinberg equilibrium was assessed, with a statistical significance level of 0.01. A chi-square test was performed to assess evidence for association. The statistical significance level was adjusted by Bonferroni correction. Linkage disequilibrium (LD) patterns and haplotype blocks were defined using Haploview 4.1.

## Results

We genotyped six SNPs that tag the majority of *CFD* haplotypes and investigated allelic association with AMD. Four SNPs, rs1683564, rs35186399, rs1683563, and rs3826945, located either at the promoter region, exon, or intron of *CFD*, exhibited no association with advanced AMD in our study population. The SNP rs34337649, in exon 5 of *CFD*, showed no polymorphisms in our data (Table 1).

**Table 1:** Association between 5 single nucleotide polymorphisms (SNPs) on complement factor D (*CFD)* and advanced age related macular degeneration (AMD)

**SNP**	**Minor allele**	**Frequency in case**	**HWE in case**	**Frequency in control**	**HWE in control**	**Frequency in HapMap**	**p (allelic)**
rs1683564	G	0.424	8.96E-01	0.409	1.57E-01	0.460 (CEPH)	0.710
rs35186399	A	0.006	9.97E-01	0.003	9.99E-01	N.A.	0.658
rs1683563	G	0.371	3.47E-01	0.414	8.21E-01	0.353	0.253
rs3826945	G	0.346	9.18E-01	0.309	9.68E-01	0.351	0.318
rs34337649	T	0	N.A.	0	N.A.	N.A.	N.A.

Shown are minor alleles for each SNP on *CFD*; frequencies for our advanced AMD, control groups and HapMap European groups; Hardy–Weinberg equilibrium (HWE) test; and P(allelic) values. (The P(allelic) value cannot be calculated when there is a count equal to zero.)

Of the six SNPs, rs1651896, which is located at the 3′ region of *CFD*, was found to be marginally associated with advanced AMD (P(allelic)=0.07, risk allele A: 37.4% in cases versus 30.7% in controls) in a Caucasian cohort of 178 advanced AMD cases and 161 normal controls. The result was further investigated in an independent replication cohort of 445 advanced AMD patients and 190 controls. No further significant association was observed for either the replication cohort (P(allelic)=0.882, risk allele A: 37.2% in cases versus 37.6% in controls) or the combined cohort (P(allelic)=0.223, risk allele A: 37.2% in cases versus 34.5% in controls, Table 2).

**Table 2:** Association between complement factor D (*CFD)* single nucleotide polymorphism (SNP) rs1651896 and advanced age related macular degeneration (AMD) in the discovery, replication and combination cohorts

**Cohort**	**Phenotype**	**N**	**MAF (A)**	**HWE**	**p (allelic)**
Discovery	Advanced AMD	178	0.374	4.21E-01	0.07
Cohort	Control	161	0.307	9.97E-01	
Replication	Advanced AMD	445	0.372	9.52E-01	0.882
Cohort	Control	190	0.376	8.41E-01	
Combination	Advanced AMD	623	0.372	9.05E-01	0.223
Cohort	Control	351	0.345	9.21E-01	
HapMap-CEU	Control	112	0.321		

Shown are the corresponding numbers of the *CFD* SNP rs1651896 for both advanced AMD and control groups in the discovery cohort, replication cohort, and combination cohort. Frequencies for the minor allele in our cohorts and HapMap European group, hardy Weinberg equation (HWE) test, and P(allelic) values are given.

**Figure 2:**
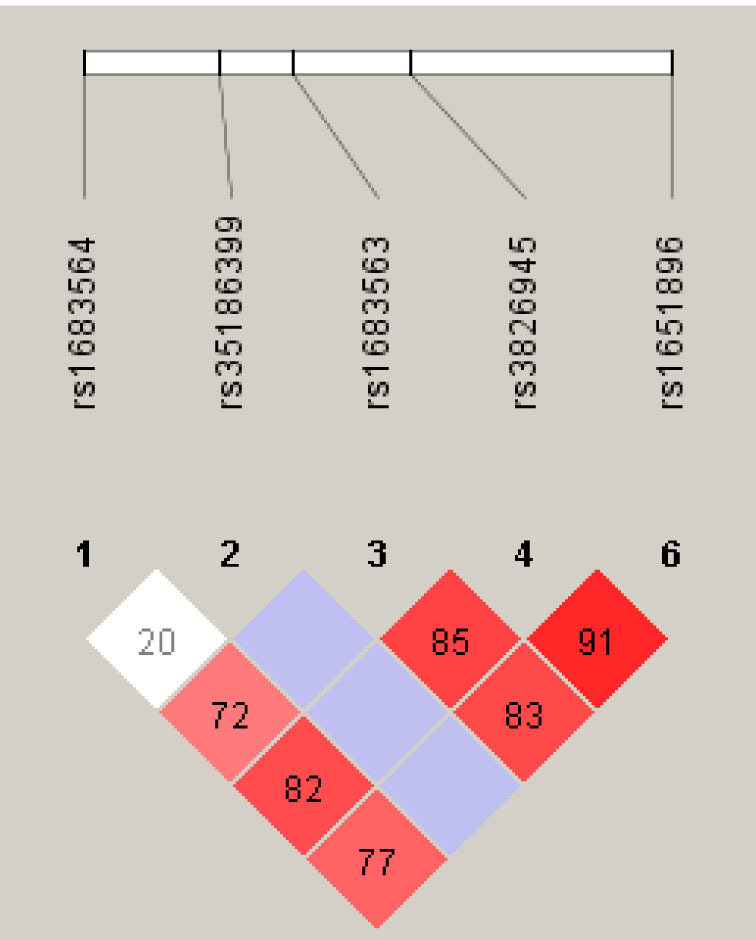
Linkage disequilibrium (LD) plot of six single nucleotide polymorphisms (SNPs) on complement factor D (*CFD).* The physical position of each SNP is shown above the plot. Darker shades of red indicate higher values of the LD coefficient (D'). The numbers in pink and white squares show the % of D' between SNPs with incomplete LD. Blue squares with no number indicate a pairwise linkage disequilibrium of 1 between SNPs, supported by logarithm (base 10) of odds (lod) scores < 2.

Linkage disequilibrium patterns and haplotype blocks were defined by combining the six SNPs (Figure 2). No significant association was found between the haplotypes and AMD phenotypes. The SNP rs34337649 was not shown in the plot because it was not a detected polymorphism in our data set.

## Discussion

**Figure 3:**
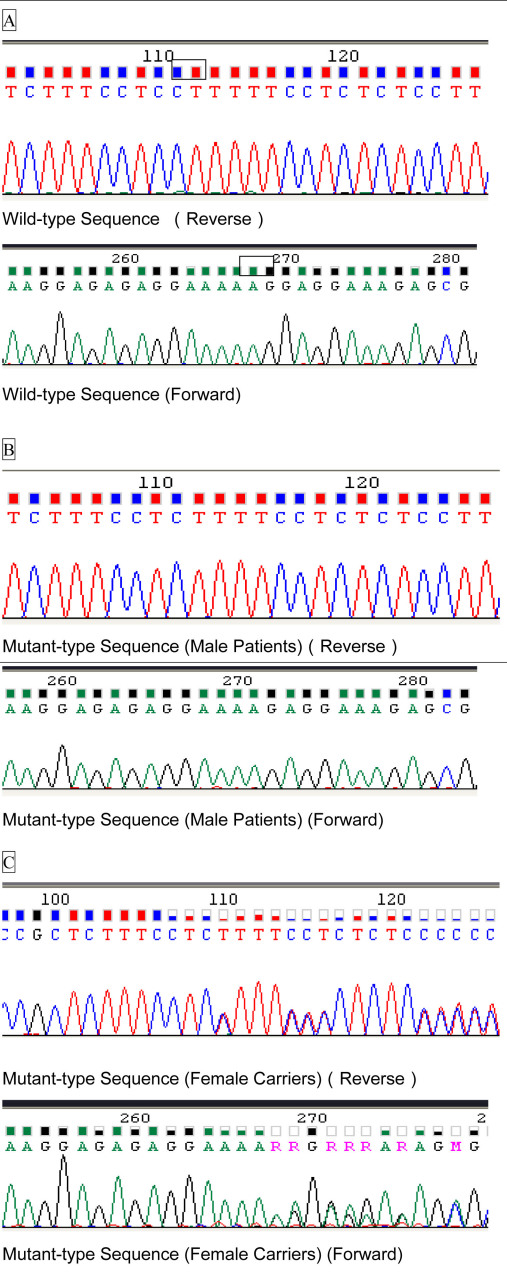
The alternative complement pathway. The principal components of the alternative complement pathway from C3 to the stage of membrane attack complex (MAC) formation are shown in detail. Complement factor H (CFH) with the dotted arrow acts as an inhibiting regulator while Complement factor B (CFB) and Complement factor D (CFD) with the filled arrows are activators.

The alternative complement pathway is one of three distinct complement pathways and is important in the clearance and recognition of pathogens in the absence of antibodies [29] (Figure 3). It is triggered by spontaneous C3 hydrolysis to form C3a and C3b, which makes C3b capable of binding to a pathogenic membrane surface. After binding with an activator membrane, C3b is bound by CFB to form C3bB. Complement factor H acts as one of the primary regulators by inhibiting the binding of CFB to C3b and also by degrading C3b. In the presence of CFD, C3bB is cleaved into C3bBa and C3bBb (C3 convertase). After hydrolysis of C3, C3 convertase and C3b become C3bBbC3b, which cleaves C5 into C5a and C5b. A membrane attack complex (MAC) is formed through subsequent reactions, which inserts into the cell membrane and initiates cell lysis [18,30,31]. In this pathway, CFB and CFD are involved in the activation and amplification loop, whereas CFH is a fluid phase inhibitor. To keep the complement activity under control, the competition between CFH and CFB binding to C3b on the host or pathogen cells needs to be tightly regulated. How this control is achieved is not completely clear [18].

Compared with other complement factors, CFH, C3, and CFB have been associated with AMD in many independent studies. However, the exact role they play in the pathogenesis of AMD and whether their effects are mediated through the complement system or through another pathway is poorly understood. Previously, CFD was studied using a *CFD* ^−/−^ mouse model, with the conclusion that eliminating the alternative pathway was neuroprotective and reduced photoreceptor susceptibility to light-induced damage [25]. Contrary to this finding, no significant association between *CFD* SNPs and advanced AMD was found in this study. Due to the dearth of information about the relationship between *CFD* and AMD, further research in other populations may be warranted. Our results suggest it is unlikely that *CFD* is a major functional candidate gene conferring risk for AMD. However, due to the relatively small sample size, this study has limited power to detect minor effects. Extended cohorts will be needed to confirm the genetic role of *CFD*. Future research should also focus on a comprehensive understanding of genetic variants throughout all pathways of the complement system. By identifying high risk variants and improving our understanding of the complement system, early intervention for patients at risk of developing AMD and novel gene-based treatments may become a reality.
